# Management of Juvenile Osteochondral Fractures Utilising Absorbable PLGA Implants

**DOI:** 10.3390/jcm13020375

**Published:** 2024-01-10

**Authors:** Hermann Nudelman, Aba Lőrincz, Anna Gabriella Lamberti, Marcell Varga, Tamás Kassai, Gergő Józsa

**Affiliations:** 1 Division of Surgery, Traumatology and Otorhinolaryngology, Department of Paediatrics, Clinical Complex, University of Pécs, 7 József Attila Street, 7623 Pécs, Hungary; nuhwaao.pte@tr.pte.hu (H.N.);; 2 Department of Thermophysiology, Institute for Translational Medicine, Medical School, University of Pécs, 12 Szigeti Street, 7624 Pécs, Hungary; aba.lorincz@gmail.com; 3 Department of Pediatric Traumatology, Péterfy Hospital, Manninger Jenő National Trauma Center, 17 Fiumei Street, 1081 Budapest, Hungary

**Keywords:** osteochondral fracture, absorbable, PLGA, osteosynthesis, articular fracture, OCF, articular congruency

## Abstract

The incidence of articular injury, particularly osteochondral fractures (OCFs), has seen a cinnotable increase in recent years. Regardless of their location, fragments can be overlooked by plain radiographs, which might lead to osteoarthritis in the long run. Diagnostic imaging has a pivotal role in the assessment and classification of the fracture severity, as well as the presence of any associated dislocations. These fractures require surgical intervention for the restoration of joint function and the reduction of long-term complications. This paper aims to present the surgical correction and post-operative treatment of osteochondral fractures with absorbable implants in four children. The following affected areas are discussed: lateral condyle of the femur, patella and radial head. Utilising absorbable implants for the management of OCFs provides numerous advantages, including the elimination of the need for re-anaesthesia and reoperation, reduction of complications and facilitation of early rehabilitation. This approach also minimises the period of hospitalisation and proves effective in pediatric OCF treatment.

## 1. Introduction

Juvenile osteochondral fractures (OCF) represent around 1–10% of fractures, and their incidence is on the rise [1,2]. This condition is characterized by the separation of subchondral bone and the articular cartilage from the articular surface; it may only involve the articular cartilage, in which case it is known as a chondral fracture. OCFs have a higher incidence in boys, with a prevalence 2–4 times greater than in girls; it is most frequently observed between the ages of 10–20, with a prevalence ranging from 8–30 per 100,000 cases [1,2,3]. The condition usually affects young athletes or children who lead an active lifestyle. Typically, OCFs arise due to traumatic events or rotational torsion of the joint, and they present acutely. Most commonly, they are the result of low-energy traumas or sports injuries [2,3,4,5,6]. The existing literature primarily emphasises chronic conditions, specifically osteochondritis dissecans (OCDs) or osteochondral lesions that develop over time. OCDs were initially documented by Konig in 1887, addressing the presence of loose fragments within the knee joint [7,8,9,10], first thought to occur due to significant trauma, repetitive microtrauma or spontaneous necrosis. However, nowadays the term refers to an area of necrosis of the subchondral bone, which may or may not be separated from the articular surface [7,8,9,10]. In this report, we will concentrate on OCFs in an acute setting.

Osteochondral lesions are associated with joint instability, resulting in abnormal motion yielding rotational, impaction, or shearing forces [1,3,4,11]. They are also often associated with sprains and dislocations, frequently in the elbow, ankle, knee (femoral condyles and patella) and hip joints [11]. At the knee joint, anterior cruciate ligament (ACL) injuries account for less than 1% of cases; however, it was found that the incidence of OCFs might be as high as 80% in the case of ACL injuries [1,11,12,13]. This is attributed to the valgus stress, which results in shearing forces that damage the cartilage of the femoral condyles [1]. The incidence of OCFs was found to be 70% in cases of patellar dislocation. As the patella shifts laterally, shearing forces act on the medial patellar facet and the lateral femoral condyle. MRI studies have estimated the incidence of OCFs in the case of lateral dislocations to be between 40% and 78% [11,14,15,16,17,18,19]. Diagnosis remains challenging, even with a comprehensive physical exam and X-ray imaging, as symptoms are often concealed by damage to the local tissue and lack specificity. Pain is the predominant symptom; it intensifies during or after weight-bearing or activities. Mechanical symptoms, such as locking or catching, are typically induced by loose fragments within the joint space. Swelling and a reduced range of motion (ROM) are typical signs. In skeletally immature patients, hemarthrosis is highly indicative of OCF. However, given the potential for acute disability due to concomitant ligamentous injury, minor pathologies or more severe injuries, a thorough diagnostic evaluation is imperative [1,11,13,14,18].

As the inherent capacity of the articular cartilage for self-repair is limited, it is critical to ensure appropriate and timely management to prevent the development of secondary osteoarthritis. Embedded within the extracellular matrix, chondrocytes make up about 1% of the cartilage volume [20,21,22]. Primary components of the matrix include collagen, water and proteoglycans. Type II collagen forms a dense network of fibres which anchor proteoglycans in place. Proteoglycans constitute 5–10% of the matrix and are formed by a protein core with attached glycosaminoglycan chains [11,20,21,22,23]. The three principal forms are dermatin sulfate, chondroitin sulfate and keratin sulfate. These molecules serve to provide structural rigidity to the cartilage through the following mechanisms:

(1) A hydrophilic nature, which pulls in water.
(2) A strong negative charge, yielding the Donnan osmotic effect.
(3) A swelling pressure provided by dense structure within the matrix, which results in strong repulsive forces [11,20,22].

Hyaluronate, another glycosaminoglycan, acts as an anchor, binding proteoglycans concurrently as hyaluronic acid. Chondrocytes, responsible for extracellular matrix production, are stimulated by mechanical load. However, their ability to divide diminishes after skeletal maturity and their numbers are downsized with age [22,24].

Biodegradable polymers were developed in previous decades for medical purposes; they include poly-glycolic acid (PGA) and poly-lactic acid (PLA) [24,25,26]. These degradable polyesters derive from monomers known as lactide and glycolide [27,28,29]. The newer generation of absorbable implants are made from poly L-lactide-co-glycolic acid (PLGA). This biocompatible polymer can be sterilised by gamma radiation, which provides sterility and reduces the molecular weight of the implant. PLGA offers a distinct advantage for controlled bio-absorption of the implants over a span of two years. The oriented PLGA’s structure prevents premature breakage and the formation of sizable, jagged or sharp-edged fragments. In vivo, degradation gives rise to minute angular or spherical particles, which slowly disappear along with a mild inflammatory reaction. The speed of degradation depends on the size of the implant and its molecular properties, which themselves primarily depend on the ratio of copolymers within the implant [26,27,28,29,30]. Copolymerisation addresses the issue of PGA degrading too rapidly and PLA too slowly. Our implants have a ratio of 85 L/15 G.

Degradation primarily occurs through hydrolysis. Secondarily, non-specific enzyme pathways also contribute to the bio-absorption process, albeit to a lesser degree. Absorption via hydrolysis gives rise to intermediary products such as glycolic acid and lactic acid. Subsequently, these intermediary products are then metabolised by the body to produce carbon dioxide and water, which are exhaled and excreted [26,27,28,29,30]. Prior to application, the implant exhibits visual transparency and slight malleability. From the point of implantation up to sixth months thereafter, hydrolysis initiates, which can be seen as a decline in molecular weight and strength over time. In vitro, the appearance transitions from transparent to whitish during hydrolysis, signifying proper degradation. After the initial six months, the implant retains its solidity; however, fragments can be broken off with substantial force. In vivo, a fortnight after being implanted into rabbit cranium, histological analysis reveals modest micro-vascularisation, along with fibroblast and osteoblast activity at the perimeters. By the 24th week, implants are predominantly fragmented, accompanied by notable osteoblast activity in the vicinity and macrophages in the surrounding area [24,25,26,31]. The implants maintain their mechanical strength and properties for a duration of at least eight weeks and eventually undergo complete absorption in about two years [24,27,28,30]. An attribute of these implants that makes them exceptionally suitable for clinical scenarios is their ability to expand in diameter and contract in length by approximately 1–2% during the initial postoperative weeks. These effects stem from water absorption and structural relaxation of the molecular arrangement. Thanks to these dimensional alterations, the implant is locked in place effectively. These characteristics provide intimate contact and firm compression of the fracture line [24,26,27,28,29,30]. One disadvantage of PLGA implants without tricalcium-phosphate tips (TCP) is that they are nearly invisible on X-ray. However, they are compatible with MR imaging.

The report aims to describe the diagnosis, management and rehabilitation of osteochondral fractures utilising bio-absorbable PLGA implants, without the need for a second surgery. The paper aspires to add to the literature as there is little written about the diagnosis and treatment of OCFs in acute settings. The following affected areas are discussed: lateral condyle of the femur, patella and radial head.

## 2. Materials and Methods

The clinical application of the technique was accepted and permitted in 2019 by our medical review board, the Hungarian Pediatric Trauma Committee, and the Hungarian Pediatric Surgery Committee. The work was performed in Pécs and Budapest at the Surgical Division, Department of Paediatrics, Medical School, University of Pécs, 7 József Attila Street, Pécs, H7623, Hungary, and at the Department of Paediatric Traumatology, Péterfy Hospital, Manninger Jenő National Trauma Center, 1081, 17 Fiumei Street, Budapest, Hungary.

Prior to undergoing general anaesthesia, all patients received antibiotic prophylaxis (Cefazolin). All operations took place in exsanguinated conditions with the patient in a supine position, and all patients received postoperative anticoagulant therapy (Clexane), as is routinely used. In all cases, the implants used were ActivaNail^TM^, produced by Bioretec, based in Tampere, Finland. The application of these implants requires drilling through the fracture with a K-wire of corresponding diameter. Sufficient depth is essential in order to avoid protrusion. The implant is then removed from its container by pushing the applicator piston into the nail head until attached. The piston is then inserted into the applicator sleeve and the nail is then introduced into the hole by sliding the piston through the sleeve and into the hole. The nail is completely inserted by lightly tapping on the piston with a mallet. The sleeve is designed to sink the nail 1–2 mm below the cortical surface to prevent irritation. To detach the nail head, the piston is rotated and pulled out of the sleeve [32]. Sinking the nail is of utmost importance to avoid irritation of the surrounding tissue and to preserve functionality; hence, if the nail is too long and protruding, hot wires or an oscillating saw can be used to cut the nail and then push it below the surface. In all cases, we followed the principles of AO fixation; primarily, we moved perpendicular to the fracture line and tried to place nails at divergent angles for greater stability. We took great care not to injure the chondral surface of the fragments any more than drilling required. The post-operative care protocol can be viewed in Section 3 of this article.

**Figure 1:**
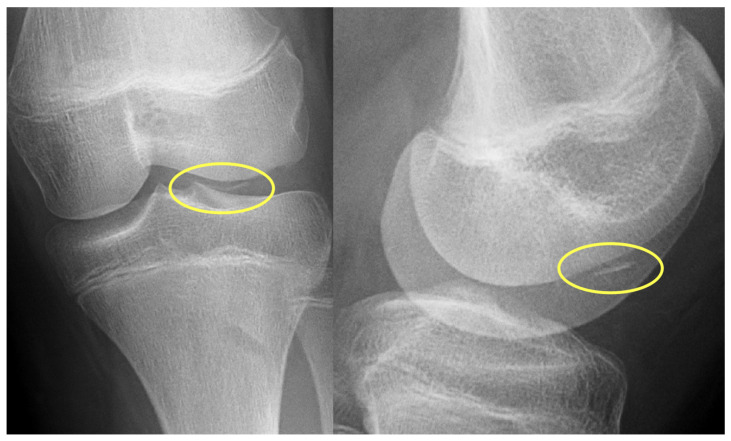
Preoperative X-ray showing the osteochondral fragment (yellow circle) of the lateral condyle of the femur.

First patient: A nine-year-old girl injured her knee during a careless step, resulting in a sprain. The left knee was slightly swollen and the patient complained about pain, but other pathologies could not be identified with the physical examination. Therefore, an X-ray was requested. Results revealed an approximately 8 mm large broken piece of the femur’s lateral condyle, positioned at the border of the lateral recess (Figure 1). 

**Figure 2:**
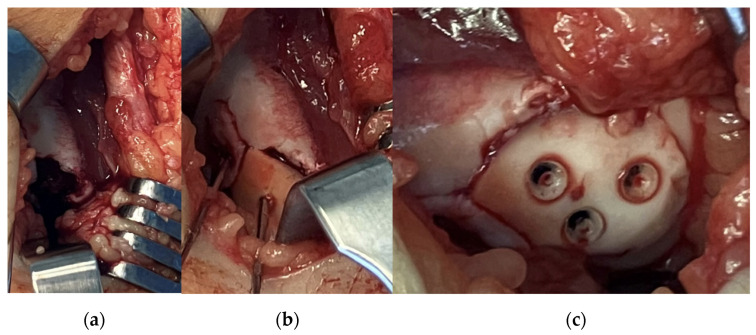
Intraoperative pictures showing (**a**) the defect, (**b**) fixation of the fragment with K-wires and (**c**) the stabilised fragment with absorbable nails.

After arthroscopy, a lateral arthrotomy was performed. The defect and the fragment were covered by fibrin coagulum, which was removed with the help of a Volkmann spoon to yield a fresh spongious surface. Then, the fragment was repositioned with the aid of three 1.5 mm K-wires. The piece was stabilised to its proper position using three 1.5 × 15 mm resorbable nails with the above-mentioned technique. After applying a drain, the joint cap and subcutaneous layers were reconstructed (Figure 2). We closed the skin incision by applying a continuous intercutaneous suture with 2/0 Vicryl. Before brace application, we performed swathing and elastic bandaging with Bactigras, in a 15-degree position.

**Figure 3:**
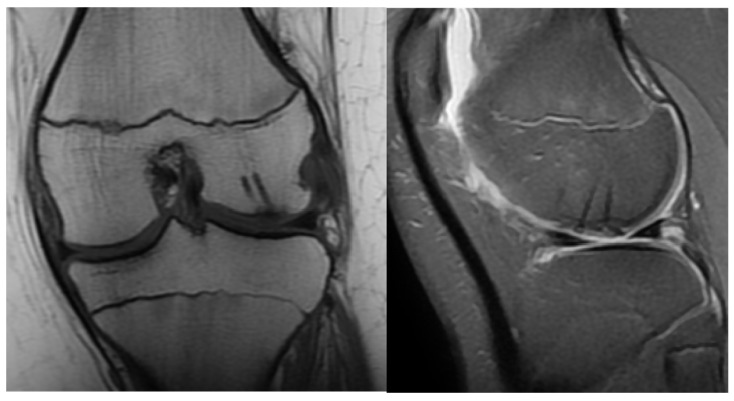
Control MRI one year after surgery. The remainder of the absorbable nails can be seen inside the femoral condyle.

The following day, the drain was removed, and physiotherapy was started immediately after the operation by CPM. A brace was worn for six weeks with a body-weight-bearing starting at zero and increasing throughout the recovery period gradually until 100% capacity. Control examinations revealed good functional results with full and pain-free extension and flexion. The X-rays showed an ideal position of the fragment at the 10th day control and on weeks 3, 6 and 12. Control MRI was performed one year after surgery (Figure 3).

**Figure 4:**
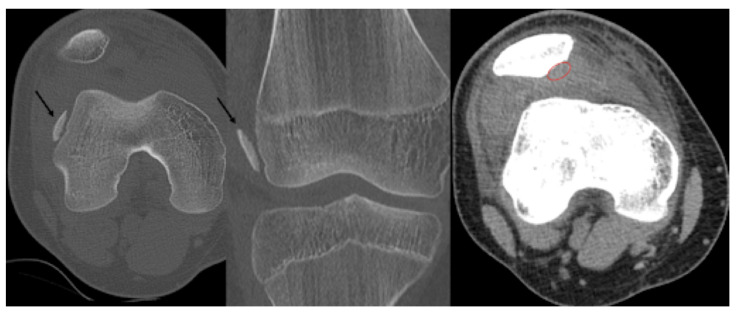
CT images of the knee, showing the fragment (black arrow) and the location of the missing piece (red circle).

Second patient: A fifteen-year-old boy was admitted after he suffered a fall. The patient complained of pain and difficulty with weight-bearing. Knee extension was painful during the physical examination, and a hematoma could be felt in the suprapatellar bursa. Primary X-rays showed the osteochondral fracture of the patella, which was confirmed by CT imaging, with the broken piece in the lateral recess (Figure 4). 

An arthroscopy of the right knee was performed to identify the missing piece and exclude any associated injuries. After medial arthrotomy, the patella was repositioned, and the broken piece became visible on the lateral aspect of the knee joint, in a close relationship to the tibial condyle. The fragment and patellar defect were covered by fibrin, so the coagulation was removed to yield a fresh spongious surface with the help of a Volkmann spoon. 

**Figure 5:**
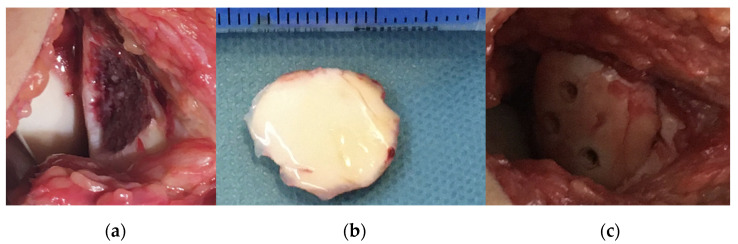
Intraoperative pictures showing (**a**) the defect of the patella before reduction and fixation of the osteochondral fragment and the fragment (**b**) after removal and cleaning and (**c**) after stabilisation with absorbable implants.

The approximately 2 × 2 cm piece was repositioned with three 1.5 mm K-wires—a proximal, a distal and a central slightly medially—drilled until the opposing cortical surface was reached. After measurements, three 1.5 mm thick and 15 mm long absorbable nails were applied successfully for desired stability (Figure 5). The capsule and the medial patellofemoral ligament were reconstructed before complete closure with 2/0 Vicryl continuous intercutaneous suture. Swathing and elastic bandaging took place before the application of a brace, in a 15-degree position.

**Figure 6:**
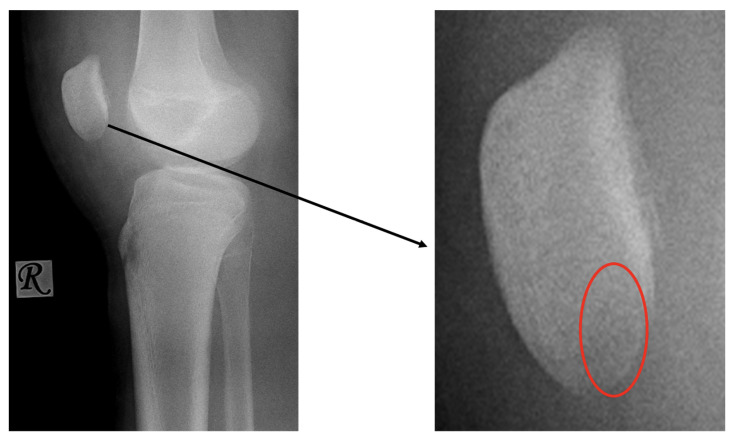
Postoperative X-ray presents the proper position of the osteochondral fragment of the patella (left image) on which the nails (red circle) are difficult to visualise.

The patient was hospitalised for two days and received physiotherapy one day after surgery in the form of CPM. Weight-bearing was limited at first, with a gradual increase throughout the recovery period until week 4. Four weeks after the operation, the weight-bearing capacity was near 100% and the patient could walk without a limp, with full extension and flexion. A control X-ray was performed in the control examinations on day 10 and weeks 3, 6 and 12 (Figure 6).

**Figure 7:**
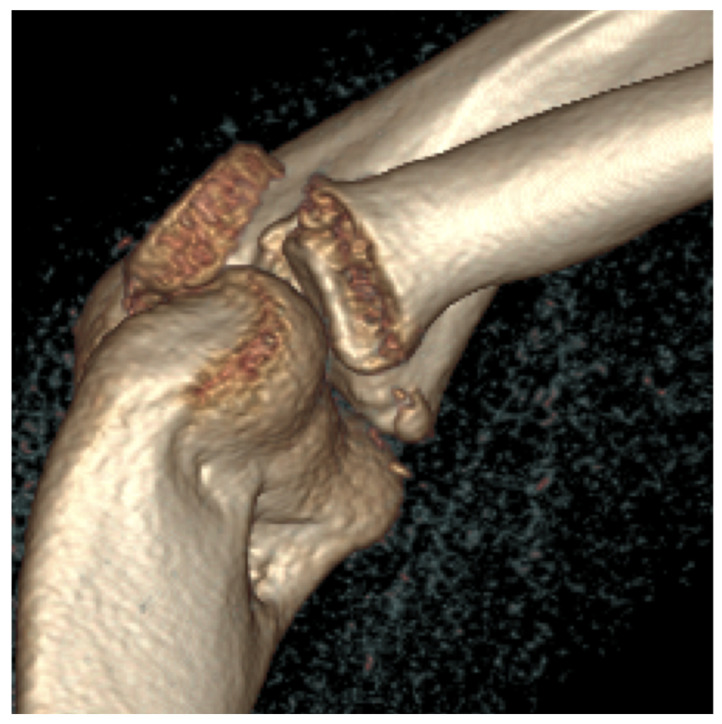
The CT images confirm the Mason type II fracture and dislocation of the radial head.

Third patient: A 17-year-old patient was admitted following a snowboarding injury that affected her elbow. Although the patient reported pain and swelling of the proximal forearm was visible upon inspection, the Moberg test was negative. The fracture of the radial head was confirmed by the initial X-ray, after which a CT was performed, which established a Mason type II fracture and dislocation of the radial head (Figure 7). 

**Figure 8:**
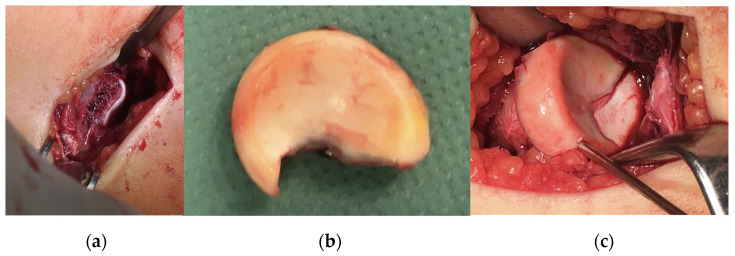
Intraoperative images of (**a**) the defect, (**b**) the fragment and (**c**) the results of the correction.

After disinfection and draping, a 4 cm incision was applied radially just above the radial head with consideration for the neurovascular structures whilst heading towards the joint cap. The fracture line could be seen clearly by passing the fascia and the joint cap, while the radial head fragment could be seen in a lateralised position. The fragment was temporarily placed into a wet dressing, the spongious surfaces refreshed with a Volkmann spoon and the fragment repositioned with three 1.5 mm K-wires; finally, it was stabilised with four absorbable nails after ROM testing (Figure 8).

**Figure 9:**
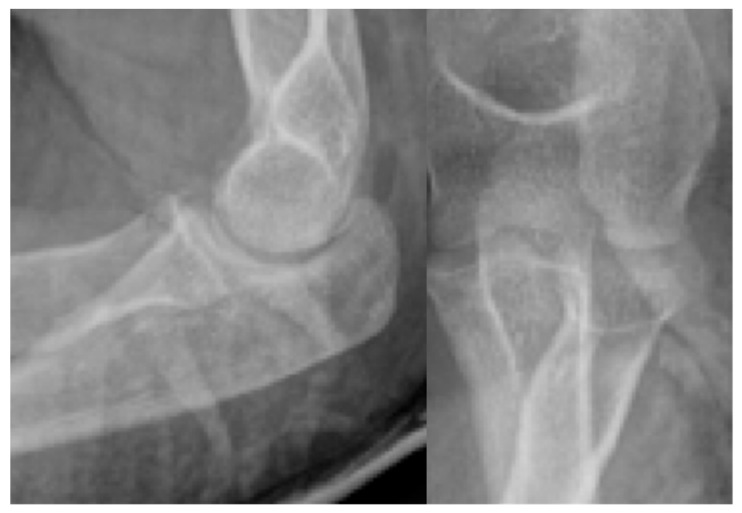
Postoperative X-ray showing the perfect position of the radial head.

The stability was confirmed by pronation and supination without dislocation, after which the joint cap was reconstructed. A 90° dorsal cast was applied with swathing and Bactigras-containing bandages, and the patient was discharged after one day. They received physiotherapy in the hospital and continued exercise training at home. Swelling and non-reactive scars were noted during the 1-week control examination; this slowly disappeared over the course of the next few weeks. A month after surgery, follow-up X-rays demonstrated the correct positioning of the radial head without any articular surface incongruence (Figure 9).

**Figure 10:**
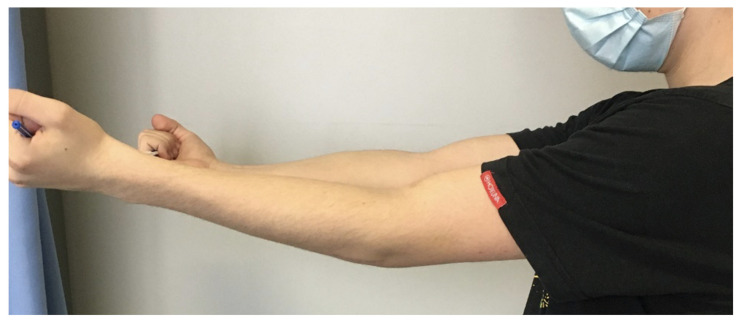
Post-op image six weeks after surgery, showing the 15-degree shortcoming of the affected side during extension.

Six weeks later, the control X-ray revealed no signs of complications; however, the affected side was missing 15 degrees of extension at this point (Figure 10). With further physiotherapy over time, the extension was improved and the joint movements were not limited in any way, being very similar to the opposing side by week 12.

Fourth Patient: An 11-year-old girl suffered a fall injuring her left elbow. During physical examination on the following day, swelling and bruising could be seen. Initial imaging with plain radiography revealed the fracture of the lateral epicondyle of the humerus with dislocation. CT imaging was requested for surgical planning and revealed the involvement of the capitulum and the trochlea. The surgery took place in exsanguinated conditions; it totalled 100 min. After disinfection and draping, we performed a radioventral approach just above the elbow. The capsule could be accessed ventrally, after passing through the brachioradialis muscle. By making a longitudinal incision on the capsule, we allowed for the hematoma to clear, which we washed several times with physiological saline for better visualisation. After that, the upward dislocation of the capitulum humeri could be seen clearly. At this point, it is noted that the opening was too small for proper visualization and handling of the fragment; therefore, the incision was lengthened distally and to the ulnar side, leaving us with an S-shaped traditional approach. The biceps tendon and the brachial artery were held out of the way with the aid of equipment to avoid damage and disturbance from repositioning. At this point, the fracture affecting the trochlea became visible. Following repositioning, we drilled three holes with the aid of K-wires for one 1.5 × 20 mm and two 1.5 × 25 mm absorbable nails. Two nails were placed in the capitulum humeri and one into the trochlea to reach final stabilisation. Before closure, we tested for the passive extension and flexion of the elbow joint, which was performed to full ROM without any notable interference. The capsule was then reconstructed and the exsanguination was stopped. The brachioradialis was then inspected and no signs of major bleeding were identified. Subcutaneous layers were reconstructed with absorbable 3/0 thread while the skin was reconstructed with 4/0 non-absorbable thread and an intracutaneous approach. After the application of bandaging with Bactigras, a 90-degree dorsal cast with a U-splint was placed on the left arm; this was utilised for four weeks. The post-op period was uneventful; neurovascular complications could not be identified; and one day after surgery, radiological findings showed the proper position of the fragment. Thus, the patient was returned to their home. Swelling and non-reactive scars were noted during the 1-week control examination; these slowly disappeared over the course of the next few weeks. Plain film radiographic controls were performed at weeks 3 and 6, which described the post-op state of a healing fracture with some decrease in ROM; however, after 4 months, the scar was healed but some locking was observed along with restricted ROM, with active movement possible between 70 and 110 degrees with proper capillary reflex. A CT examination was ordered, which revealed the normal healing of the capitulum humeri with a lytic lesion on the border of the capitulum and the trochlea. To improve functionality, subaquatic physiotherapy was started. In the 1-year control examination, the patient described better functionality with active motion of 40–110 degrees with continued physiotherapy. Control CT was performed after 2.5 years; it revealed the appropriate position of the elbow joint without any pathological findings or lesions.

## 3. Discussion

Presently, the existing literature indicates that OCFs have a higher incidence in children, who live active lifestyles involving sports, dancing, gymnastics and so on. Research has shown that the pediatric age group is more susceptible to OCFs due to their lower resistance to sheer forces [1,13,33,34]. As previously discussed, diagnosis can be challenging given the difficulty of visualising small pieces on plain radiographs, the presence of non-specific symptoms and the limited access to diagnostic tools. It is worth noting once more that symptoms such as pain, swelling, locking and catching, and hemarthrosis are common and are suggested to be positively suggestive of articular surface injury [1]. A straightforward diagnostic algorithm was proposed in a 2015 paper authored by Pedersen et al. [11]. We believe that this algorithm is adequate and can serve as a useful template when deciding upon the diagnostic evaluation of the injury. The importance of high-risk signs should not be neglected, and they should warrant further evaluation for fitting diagnosis and treatment.

Imaging serves several critical purposes, including confirming clinical suspicion, assessing the extent of articular surface damage, detecting instability or dislocations, and monitoring disease progression [1,11,18]. Following acute joint trauma, the standard examination includes plain radiographs. However, OCFs might be difficult to identify as small bony fragments cannot be visualised, as opposed to a large piece of detached articular cartilage. Features indicative of OCFs on plain radiographs include irregular bony contours, fragmentation or a thin piece of radiodense subchondral bone. According to the literature, the sensitivity of X-rays for the diagnosis of OCFs is between 32 and 69%, depending on the location. For instance, OCFs affecting the talus can be diagnosed with X-rays with a 69% accuracy, while OCFs of the knee and hip reach only 32%, as demonstrated and proven by McCarthy et al. [1,35,36]. Therefore, in acute settings, radiographs should be extensively and thoroughly searched for OCFs. Awareness should be given to prevalent locations of lesions, such as the talar dome or the lateral recess in cases of patellofemoral injuries [3,14,36].

In cases where fractures affect the articular surfaces, alternative imaging modalities are recommended, as well as for surgical planning. Computed tomography (CT) and magnetic resonance imaging (MRI) are superior to plain radiographs for the detection of OCFs. CT is particularly effective in identifying small osteochondral fragments and thin bony plates with high-resolution images. However, it cannot depict bone marrow oedema. As an addition, CT angiography can reveal small chondral pieces as well. MRI is also able to detect chondral injuries with a high sensitivity and specificity (depending on the region) of about 75–93% [37,38,39]. A study observed and noted that the sensitivity of MRI is slightly higher than that of CT, but this difference is not statistically significant. A potential advantage of MR imaging is that it can detect other soft-tissue abnormalities and injuries which are often associated with OCFs. This can be particularly beneficial in cases concerning sprains and ligament or tendon abnormalities which may contribute to continued instability and pain [1,37,38,39]. MRI findings indicative of lesion instability include the presence of fluid signals between the fragment and its origin. Other indications of fragment instability include the collapse of the articular surface, extensive bone marrow oedema and cystic changes [1,36,37,38,39]. Contrast material, when positioned between the fragment and the subchondral bone, can also serve as an indicator of instability, detachment or dislocation. Consequently, both CT and MR arthrography are sufficient to assess the instability of the fracture and to achieve accurate staging [11,36,37,38,40].

The staging system for osteochondral lesions can be based on various imaging modalities, including X-ray (Berndt and Harty), CT (Ferkel), MRI (Happle) or arthroscopic (Cheng–Ferkel) in cases affecting the talus [38]. Nevertheless, depending on the size and location of the fragment and the amount of bone involvement, radiographs might not be able to show loose bodies. CT and MRI attempt to grade the stability and severity of the injury and are further utilised for surgical planning. A summary of these assessments can be seen below:

A. Stage I:
  a. Injury limited to articular cartilage
  b. Subchondral edema on MRI
  c. X-ray: negative
B. Stage II:
  a. Cartilage damage with subchondral fracture without detachment
  b. Thin sclerotic margin
  c. X-ray: negative or slight sclerosis
  d. Subtypes:
    1. Type A: cystic on CT and oedema on MRI
    2. Type B: non-displaced and incompletely undercut by fluid (MRI) and lucency (CT) with an open connection to the articular cartilage.
C. Stage III:
  a. Detached, non-displaced
  b. MRI: rim-sign—high signal around fragment
  c. X-ray: lucency between fragment and normal bone
D. Stage IV:
  a. Osteochondral fragment is displaced
  b. Joint effusion
  c. X-ray: loose body, lucency
E. Stage V:
  a. Subchondral cyst formation
  b. Secondary degenerative changes
  c. X-ray: secondary osteoarthritis

Traditionally, the management of fractures affecting the articular surface necessitates osteosynthesis of the fragment via various approaches. Typically, OCFs are to be treated operatively [1,34]. The literature discusses the utilisation of several fixation methods, such as the use of Herbert screws, compression or headless compression screws, magnesium screws, meniscus arrows, bone tunnel sutures or bioabsorbable screws and pins [1,15]. In specific cases, when the fragment is too small for fixation (usually less than 5–10 mm) or if the fixation is otherwise contraindicated by degenerative conditions, it might be necessary to remove the fragment and address the defect with cartilage restoration methods. Restorative options encompass debridement, autologous chondrocyte implantation, mosaicplasty, allografts and the use of biomaterials [1,11,15].

The authors would like to emphasize that in children, the appropriate management strategy for OCFs is contingent on various factors, including the placement of the lesion within the joint, the age of the child, their growth stage, size and the specific anatomical site of the defect; these all dictate the proper management strategy. This implies that a 5 mm defect might necessitate different management strategies based on whether it is located in the knee (as it is a weight-bearing surface) in contrast to the elbow. Additionally, a 5 mm defect in a small child holds greater significance compared to an almost skeletally mature patient. In these cases, fragments should be reattached to achieve optimal outcomes.

Delayed or inappropriate treatment may significantly impact quality of life. The primary objectives are to achieve a stable fixation, restore articular congruity and joint stability, and allow for early passive motion. Complications arising from implant protrusion or migration can result in injury of the surrounding meniscus, bone or cartilage. Malunion, nonunion, inflammation, infection, pain and functional deterioration can all occur in cases of malposition and are more frequent with metal implants [1,11,33,34]. Metal implants might provoke more irritation or hardware reactions such as inflammation or foreign body reactions. Biodegradable instruments are groundbreaking in this aspect as they are associated with fewer complications [32]. Consistent with other research findings, the size of the bony fragment should define the fixation tool (screw, pin, nail and adhesives), and the size of the chondral part is critical to determine the treatment method. Considering the enhanced healing capacity of the adolescent population, we suggest the operative treatment of OCFs if weight-bearing surfaces are affected, mechanical symptoms are present, diagnostic evidence can be found and the fragment is sufficient in size. We suggest that operative treatment takes place promptly for OCFs, with a particular focus on restoring articular surface congruency. It is advisable to clean the fragments of fibrin-coagulum using a curette and to create smooth edges to ensure proper attachment with the underlying fresh spongious surface without gaps or incongruency, if feasible [1,11].

Timely and appropriate treatment is vital to prevent long-term joint dysfunction, deformities and a restricted range of motion. While the chondral part of the fragment may potentially receive nutrients via diffusion from the synovium, the same cannot be said about the bony element, which exhibits a reduced ability to heal over time [1]. Nevertheless, it will retain its role as a scaffold. Bioabsorbable implants have been recommended as a suitable treatment for OCFs by Schleter et al. and Hsu et al. among many others [1,9]. Our suggestions for the indication of absorbable implants are as follows:

1. Osteochondral or chondral fragment
2. Single, isolated body
3. Sufficient size
4. Instability, detachment or movement

The authors propose the arthroscopic removal of the fragments if:

1. Fragment is less than 5 mm
2. Bodies are multi-fragmented and small

Biodegradable implants are thought to be less detrimental to the surrounding tissues and cartilage; however, they might provide less stability. An additional advantageous attribute resides in the bending modulus, which is closer to that of bone in the case of absorbable implants, in contrast to metal implants. This characteristic safeguards the fixation from adverse effects due to stress shielding [30]. Absorbable implants are less prone to interfere with growth, promote bone remodeling and lead to fewer biochemical reactions that might harm the patients’ recovery [1,11].

One issue regarding the fixation of osteochondral lesions is the fate of the articular surface and its congruency. Fibrocartilage formation may alter the function of the joint. For this reason, the nails are placed below the articular surface and CPM is started as soon as possible to ensure adequate healing; furthermore, 3D-printed PLGA implants have been used for cartilage repair with sufficient pore sizes (300–400 nm) to allow for cellular ingrowth; however, these are not available to every hospital. The absorption process of oriented PLGA implants was investigated through MRI imaging by Hedelin et al. in the hip osteosyntheses of children, and results reported that the vast majority of implants were completely absorbed and that 2 years after implantation the canals were over 90% filled with bony tissue [27]. No significant local host reactions were observed. In our control MR image, we can see proper lining of the articular surface without incongruency and bone ingrowth at the sites of absorption. Newer generation implants composed of PLGA provide a possibility for controlled absorption, which solves the issue of excessively slow degradation, which was the case in previous generations of absorbable implants and which caused irritation and metabolic disturbances [24,26,27,30], more detail on Supplementary Materials link.

The comparison of metal to PLGA implants regarding the fixation of osteochondral fractures in the literature is rare. However, studies describe good results utilising biomaterial composites (PLA, PGA, PLGA, and PLLA) in other locations, such as during ACL repair, tibio-fibular syndesmolytic breaks, intramedullary nailing of the forearm and osteosyntheses of various locations. A study that investigated the effectiveness of absorbable screws in comparison with metal screws regarding Maisonneuve fractures reported no significant differences between complication rates, ROM, dorsiflexion or plantarflexion, OM score or VAS score between the groups. In contrast to metal implants either titanium, steel, or magnesium, absorbable interference screwswith zinc led to less soft tissue irritation. A study investigating the advantages of bioabsorbable screws over metallic screws in regard to ligamentous injuries lists several beneficial properties such as no interference with surrounding tissues over a long time, no osteoporosis, less toxicity and reduced risk of infection, as well as controlled absorption in the case of PLGA implants. The study also names several disadvantages regarding the varying degradation rates depending on the material and its ratio of copolymers, effusion around the joint, rapid loss of screw strength, inadequate stiffness and inflammations that may cause osteolysis or cyst formation [41]. Metal implants are less likely to lead to fluid effusion or tunnel widening and osteolysis or cystic changes; however, these require a removal surgery, might irritate surrounding tissues and pose a risk for infection [41]. A prospective randomised study about ACL reconstruction also concluded that there were no statistically significant differences regarding functional outcomes between the two methods. A different study, however, reported increased rates of complications such as graft rupture, joint effusion or infections in regard to ligamentous injury repair [42]. A meta-analysis of syndesmolytic injuries accounts for fewer complications when using absorbable implants. The literature regarding different types of degradable and non-degradable implants was described by Gentile et al. and Al-Shalawi et al. and others in many ways, by comparing traditional metal and interference screws against several types of bioabsorbable implants for various purposes. One notable disadvantage mentioned is the production of acidic by-products at higher concentrations which can alter osteoblast activity and thereby hinder healing around the implant. To counter these adverse effects, the implants were combined with beta-tricalcium phosphate and hydroxyapatite, which are used in orthopaedics. It is difficult to discern from this study, due to the small sample size, whether PLGA implants are superior to other types of bioabsorbable materials when it comes to the repair of the articular surface; because of this, more extensive studies are needed in order to understand which implants and which properties are suitable for the correction of osteochondral fractures. There are many types of implants to choose from; however, accessibility might be difficult depending on funding and location of the healthcare provider. Not all hospitals or centres are equipped with 3D printers for the production of individualised biomaterial scaffolds with adequate pore sizes for cellular ingrowth, and materials are not necessarily readily available to everyone; thus, we propose, based on the readily available evidence, that commercialised oriented PLGA implants may provide at least as good a result as metal counterparts in OCF fixation.

Rehabilitation following surgical treatment involves physiotherapy and brace use for 2–6 weeks depending on severity. Children should start rehabilitation right after surgery with the help of physiotherapeutic training [33,40]. We advise early mobilisation during the immediate postoperative period as well as continuous passive movement (CPM) and ROM exercises, beginning on post-op day 1 and lasting until day 21. Physiotherapy and active motion exercises should begin after the first week, on day 8. In the case of weight-bearing joints, we suggest that weight-bearing is limited initially with a gradual increase until weeks 6–8. We suggest a return to sports activities after 4–6 months, depending on severity. The utilisation of these techniques allows for proper healing and helps decrease the occurrence of post-op complications [1,11].

A second anaesthesia would increase cost and prolong the length of the hospital stay. Complications arising from a second surgery are eliminated. Because of this, it greatly reduces the burden on the child and healthcare provider. The overall length of the hospital stay is decreased. The expenses of the healthcare provider can be reduced as there is no need for staff, operating rooms or materials to be used at a second surgery. The child may recover from the comfort of their home and may begin physiotherapy sooner.

This study is limited by its small sample size and by its lack of (1) control for confounding variables and bias, (2) comparison with other materials and outcomes, and (3) ability to establish causality by limited standardisation. However, the real incidence, prevalence and outcome of the condition cannot be found in the literature, nor can generalised guidelines be found regarding diagnostic and treatment algorithms for OCFs. More extensive studies are needed in both pediatric and adult populations.

## 4. Conclusions

With the application of absorbable implants, the need to re-anaesthetise and reoperate was eliminated; thus, the strain on the patient was significantly reduced. Furthermore, possible risks relating to the surgery—such as infections or other complications—are also decreased. Patients may begin physiotherapy sooner due to the reasons mentioned above. The countersink option provides a frictionless environment for healing, decreasing the chance of complications due to shear forces, which are the result of protrusion or malposition. PLGA implants provide the possibility for controlled absorption and replacement of the nail with bony tissue over time. Even though the study is limited by its small sample size and its nature as a case series, it may be that PLGA implants provide at least equal—if not better—results to metal competitors in the fixation of OCFs.

## Data Availability

The data are contained within this article.

## Supplementary Materials

Information regarding the implants, their production and application, as well as other supporting information such as whitepapers, studies, operation videos and animations, can be downloaded at https://bioretec.com/educational-materials.
